# Supplementary material for: A Global Screen for Assembly State Changes of the Mitotic Proteome by SEC-SWATH-MS
Source: Cell Syst. 2020 Feb 26;10(2):133–155.e6. doi: 10.1016/j.cels.2020.01.001 (PMC7042714; doi:10.1016/j.cels.2020.01.001)

Q9BQL6 | FERM1_HUMAN | FERMT1 C20orf42 KIND1 URP1
Monomer MW [kDa]: 77.437 Monomer expected elution fraction: 42

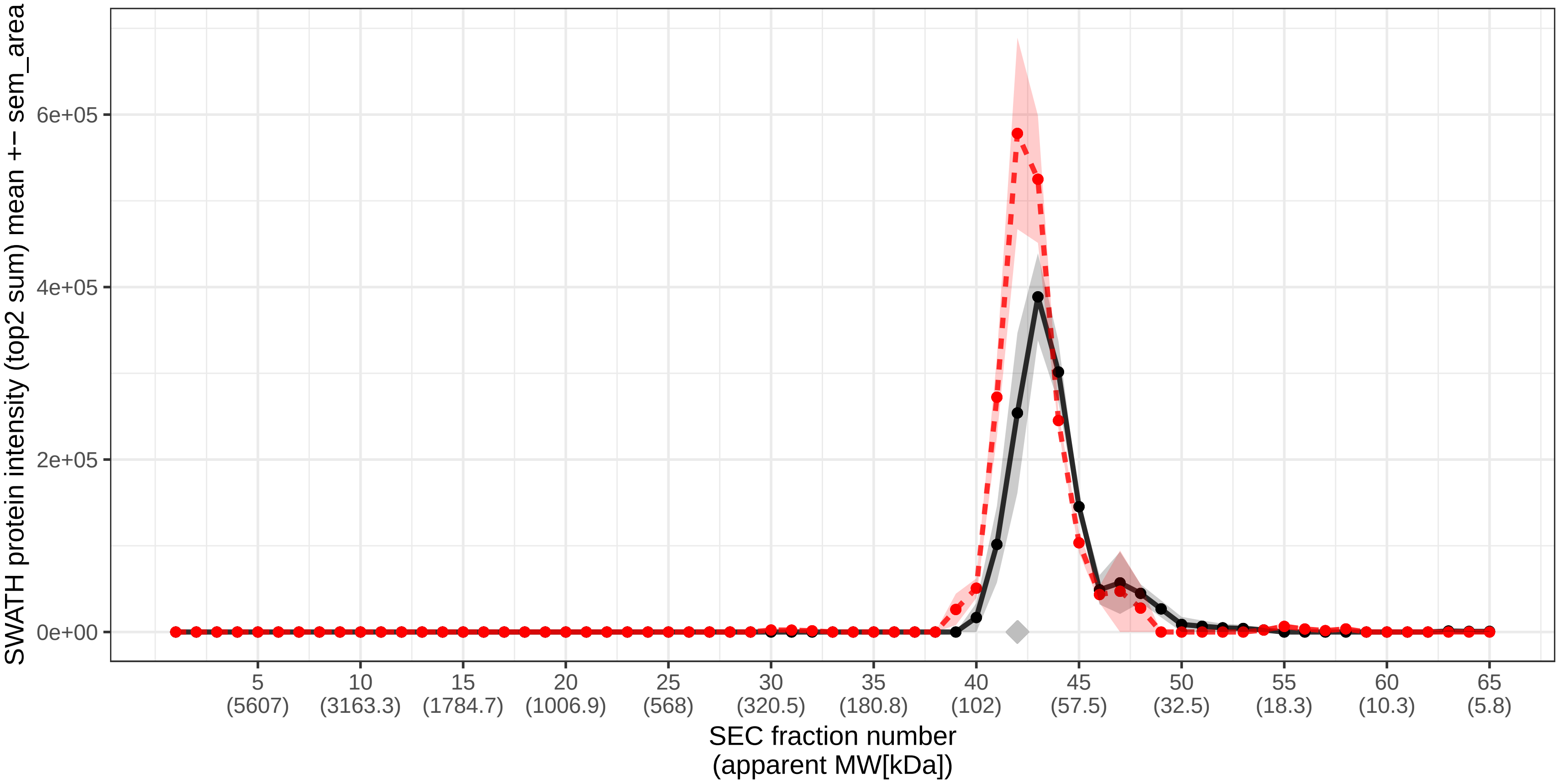

Supplement: Data S1. SEC-SWATH-MS Protein Chromatograms, Related to Figure 1 [file mmc6.zip › SECchrom_Q9BQL6_FERM1_HUMAN_FERMT1_C20orf42_KIND1_URP1.pdf]
